# Erratum to: What can ecosystems learn? Expanding evolutionary ecology with learning theory

**DOI:** 10.1186/s13062-016-0132-7

**Published:** 2016-06-20

**Authors:** Daniel A. Power, Richard A. Watson, Eörs Szathmáry, Rob Mills, Simon T. Powers, C. Patrick Doncaster, Błazej Czapp

**Affiliations:** Electronics and Computer Science, University of Southampton, Southampton, SO17 1BJ UK; Institute for Life Sciences/Electronics and Computer Science, University of Southampton, Southampton, UK; The Parmenides Found, Center for the Conceptual Foundations of Science, Pullach, Germany; Department of Informatics, Faculty of Sciences, University of Lisbon, Lisbon, Portugal; Department of Ecology & Evolution, University of Lausanne, Lausanne, Switzerland; School of Biological Sciences, University of Southampton, Southampton, UK

## Erratum

After publication of this article [1], it was noticed that equation 1 incorrectly contained an additional ‘+’ sign. The correct version of equation 1 is included in this erratum.
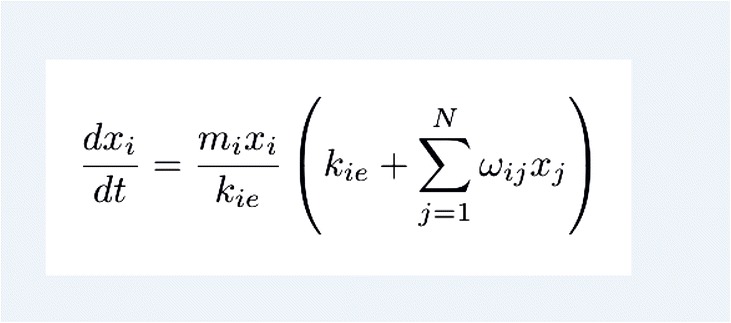


In addition, it was noticed the email address for the corresponding author, Daniel A. Power, is incorrect. This is correctly included in this erratum under the correspondence section.
